# Potential for *Saccharina latissima* Flour as a Functional Ingredient in the Baking Sector

**DOI:** 10.3390/foods12244498

**Published:** 2023-12-16

**Authors:** Oana Bianca Oprea, Ignat Tolstorebrov, Ingrid Camilla Claussen, Sigurd Sannan, Livia Apostol, Claudia Moșoiu, Liviu Gaceu

**Affiliations:** 1 Faculty of Food and Tourism, Transilvania University of Brasov, 148 Castelului Street, 500014 Brașov, Romania; 2 NTNU, Institutt for Energi- og Prosessteknikk, Postboks 8900 Torgarden, 7491 Trondheim, Norway; 3 SINTEF Energi AS, Postboks 4761 Torgarden, 7465 Trondheim, Norway; 4 SINTEF Ocean AS, Postboks 4760 Torgarden, 7465 Trondheim, Norway; 5 National Reseach & Development Institute for Food Bioresources—IBA Bucharest, 6 Dinu Vintilă Street, 021102 Bucharest, Romania; 6 CSCBAS&CE-MONT Centre/INCE-Romanian Academy, Casa Academiei Române, Calea 13 Septembrie No. 13, 050711 Bucharest, Romania; 7 Academy of Romanian Scientists, Ilfov Street, No. 3, 050044 Bucharest, Romania

**Keywords:** macroalgae supplements, bread, functional food, characteristics, sensory evaluation, seaweed

## Abstract

The healthy “superfood” sector is currently quickly developing in Europe, and grocery stores are increasingly stocking macroalgae food supplements. Due to its high amount of protein, fiber, and minerals, numerous studies have demonstrated that seaweed has a significant potential for usage as a functional ingredient in the food sector. The aim of the current study was to evaluate the rheological (ICC 173 standard method) and chemical potentials of using *Saccharina latissima* flour in the bread sector. The calcium level of S. latissima flour was found to be 8236 mg/kg, the magnesium level was 6041 mg/kg, the K concentration was 62,088 mg/kg, the iron content was 35.23 mg/kg, the P content was 2263 mg/kg, and the I content was 12,530 mg/kg, significantly higher values than those of wheat flour. The antioxidant properties of the algae powder used were highlighted by the analysis of the total polyphenol content and its antioxidant activity (DPPH method). Four bread samples, which were compared with the control sample entirely made of wheat flour in order to evaluate their potential, were made, using a replacement degree from 1.5% to 6% of *S. latissima*. Rheological analyses were completed using the ICC 173 standard method, as well as sensorial analysis, where a panel of assessors’ evaluations compared the sensory properties of samples with 1.5–6% of *S. latissima* flour to a control sample manufactured with flour type 650. It was concluded that sample A1 (1.5% algae flour) has sensorial properties similar to those of the control sample, and, for the other samples, the properties began to degrade with the increase in the amount of algae flour. Textural analyses performed during 96 h of storage show that the firmness and gumminess increase with the addition of algae flour and over time. The conclusions indicated that samples comprising 4.5% and 6% of *S. latissima* are unsatisfactory from a rheological and sensory perspective, while samples having 1.5% and 3% of *S. latissima* can be viewed as sources of fiber and minerals.

## 1. Introduction

The consumption of macroalgae, which occurs with regularity in East Asian countries such as China, Japan, and South Korea, has led to several beneficial health effects. Specifically, cases of cardiovascular diseases and neurological problems in the population have decreased, and the seaweed diet has shown to have anti-inflammatory effects [1,2,3]. In the past decades, Japanese people have achieved, due to their well-known eating habits and the consumption of seaweeds, the longest life expectancy in the world [4]. As a result, a growing interest in the production and consumption of items generated from macroalgae has been noted in Western countries in recent years. Therefore, as noted by Kumari et al. [5] and Leandro et al. [6], the inclusion of macroalgae in Western diets should affect the mineral intake of the population.

The investigation of seaweed superfoods as bioactive nutraceuticals offers a viable and comprehensive strategy to address the dietary requirements and overall health of people with hyperglycemia, diabetes, and celiac disease [7].

*Saccharina latissima*, also known as sugar kelp, is one of the most important macroalgae of the brown algae (*Phaeophyceae*) category, and it is most commonly found in the North Pacific Ocean and the North Atlantic Ocean at depths between 8 m and 30 m. Exceptionally, sugar kelp is found in warmer waters, at greater depths down to about 120 m, in the Mediterranean Sea but also in Brazil [8,9,10].

Brijesh et al. [10] provided evidence for the fact that ocean farming, compared to traditional land-based farming, is more sustainable due to the lack of chemical fertilizers and fresh water use in the cultivation of seaweeds. Also, besides the fact that seaweed is an excellent source of nutrients, seaweeds can be used in biofuel production and for animal feeds and serve as an important source for biomass.

The behavior of cultivating *S. latissima* in a coastal environment was examined in a study by Visch et al. [11], who concluded that seaweed aquaculture has limited environmental impacts, especially when compared to other aquaculture forms like fish and bivalve farming. Additionally, it was found that *S. latissima* attracted seven additional kinds of seaweed in addition to 17 species of mobile fauna, indicating that seaweed farms might guarantee the optimal environment for these species [11].

It should be highlighted that there is a high dispersion of data collection for macroalgae in terms of their ability to accumulate a specific biocompound (especially minerals). The region, seasonality, human activity, processing technology (for example, the type of water used in washing methods), and laboratory maneuvers are just a few of the variables that can cause variances in the literature data. For instance, Larrea-Marn et al. [12] discovered that samples of *Porphyra algae* taken from Spain and France had a Na content that was almost 10 times higher than samples taken from Japan and South Korea.

In order to use less sodium chloride and increase their mineral content, fortified meals made with seaweed have been the subject of several studies. Thus, López-López et al. [13,14] and Cofrades et al. [15,16] accomplished excellent work in repurposing multiple meat products, being able to substitute the NaCl addition to some extent with various edible seaweed species while keeping their tactile and sensory qualities. These studies were particularly focused on meat-based products.

For many significant geographical areas of the world, milk, dairy products, and even so-called plant “milks” (made from soy, almond, oat, or rice) constitute a significant food segment [17]. As a result, they can be suitable for macroalgae supplementation or as “vector” foods, with similar concerns being noted in other food categories like pasta, baked goods, and snacks [18]. Several concerns were registered in this area, with licenses for enriched dairy products with seaweed-derived minerals [19,20].

Consumer preferences have undergone significant change over the past few decades, from previous preferences for refined products and doughs with strong baking qualities but few fibers and minerals, in particular [19]. Thus, consumers have changed their behavior to favor more nutritionally balanced bakery items, as a result of diseases associated with past eating habits. The demand for new products drove the development of novel manufacturing techniques that have allowed for the production of nutritionally diverse products [5,6,8,10,13]. 

The goal of this research is to evaluate the potential for *S. latissima* flour to be used as a functional ingredient in the baking sector from a chemical and rheological perspective. Additionally, the maximum amount of wheat flour that can be substituted with *S. latissima* is decided by respecting the bakery’s requirements and taking into consideration the panelists’ sensory evaluations. Finally, textural analysis during 96 h of storage is made in order to determine the variation in firmness, cohesiveness, elasticity, and gumminess over time and regarding the effect of *S. latissima* addition.

## 2. Materials and Methods

### 2.1. Flour Mixtures

In the study, two types of ingredients were used from which flour mixtures were prepared: wheat flour (WF) type 650 (moisture = 13%; protein = 11%; ash = 0.65%; carbohydrate = 74%; raw fiber 1.2%) supplied by Băneasa Moara S.A., (Buftea, Romania) and macroalgae—*S. latissima* (moisture = 7%; protein = 14%; ash = 38%; carbohydrate = 35%; raw fiber = 6%) provided by Seaweed Solutions AS, Norway.

The seaweeds were deliverd in a frozen state and vacuum-packed in 2 kg low-density polyethylene bags. The frozen seaweeds were cut into small pieces (0.02 m × 0.02 m × 0.02 m) and dried in a microwave-assisted vacuum-freeze dryer μWaveVac0250fd (Püschner Microwave Power Systems GMBH, Sschwanewede, Germany). Each batch was 10.0 ± 0.5 kg. The following process parameters were maintained during the drying: vacuum in the chamber at 0.1 mbar and temperature at crystallizer −65.0 ± 0.2 °C. The microwave power was set at 1000 W at the beginning of the drying process, with a gradual decrease to 200 W for the final drying stage. 

The total drying time was between 10 and 11 h. The temperatue of the surface of the seaweeds was maintained below 20.0 ± 0.5 °C during the drying process to provide a high quality of the dried product. The dried seaweeds were then milled into powder using a laboratory blender CB15E (Waring Conair, Torrington, CT, USA) at 5000 rpm. The dried powder was sieved via a grid of 0.2 mm and vacuum-packed in LDPE bags of 1 kg. The dried powder was stored at 20.0 ± 5 °C before further processing.

Table 1 presents five samples of wheat and algae flour mixtures, with different added proportions of the seaweed flour. Comparative rheological analyses were performed for the mixtures of *S. latissima* and 650 type WF, while each of the four mixture samples was compared to a control sample (M: 100% type 650 WF).

### 2.2. Moisture Content Analysis

The moisture content was verified to be conformant to SR ISO 712:2009 [21]. The analysis was based on the thermogravimetric principle and established the mass loss by heating the mixture to 130 ± 3 °C using a Mettler LJ 16 thermobalance (LabMakelaar Benelux B.V., Zevenhuizen, The Netherlands) as a heat source. The determination of the moisture content was necessary for reporting the results of experimental analyses of a dry substance or with a standard water content. 

### 2.3. Ash Content Analysis of Studied Flours (S. latissima and Wheat Flour)

The method of determining the ash content according to SR ISO 2171:2009 [22] consists of calcination at 550 °C to decompose the organic substances in the sample to be analyzed, and then weighing the obtained ash. A thermo-adjustable electric oven, “Nabertherm” (Nabertherm GmbH, Lilienthal Germany), was used for the calcination. 

### 2.4. Protein Content Analysis of Studied Flours (S. latissima and Wheat Flour)

The protein content of the samples was established in accordance with SR ISO 20483:2007 [23]. The procedure consists of the decomposition of organic substances, in the presence of a catalyst, with concentrated sulfuric acid, the alkalinization of the reaction product, distillation, and the titration of the released ammonia. 

The nitrogen content *w_n_* is calculated from the amount of ammonia produced, and the total protein content *w_p_* is calculated from

w_p_ = w_n_ × F, (1)

where w_n_ is the nitrogen content of the sample, expressed as a mass percentage, and F is the Kjeldahl nitrogen conversion factor to protein (F = 6.25).

The following equipment was used to determine the total protein content: Tecator Digestor Auto mineralization unit (Foss, Hilleroed, Denmark), Tecator Scrubber gas scrubbing unit (Foss, Hilleroed, Denmark), Kjeltec 2300 analysis distillation system (Tecator, Hoganas, Sweden), and FOSS digestion tubes. The reagents used in the determinations were Kjeldahl catalyst tablets, sulfuric acid d = 1.83–1.84 and 0.1 n, and NaOH solution 30% and 0.1 n.

### 2.5. Total Fat Content Analysis of Studied Flours (S. latissima and Wheat Flour)

The total fat content was determined by continuous flow extraction according to SR 90:2007 [24] of the fats from the sample, at a temperature < 100 °C and with the help of an organic solvent (petroleum ether, ethyl ether, hexane, etc.). By this method, all simple or complex lipids were extracted from the product. A Soxhlet extraction setup was used for fat determination.

### 2.6. Crude Fiber Content Analysis of Studied Flours (S. latissima and Wheat Flour)

A method for the determination of crude fiber, cellulose, hemicellulose, and lignin was taken into account. The method consisted of treating the sample to be analyzed with an acid detergent solution (ADF). The ADF was made up of 20 g of N-cetyl-N,N,N-trimethylammonium bromide diluted in 1 L of H_2_SO_4_, concentration 0.5 mol/L, by using Fibretherm-Gerhardt equipment. This acid detergent solution was used because the cellulose and lignin in the structure of the material to be analyzed do not dissolve in it, unlike the other components. Using special bags for fibers (FibreBags), the dilution and filtration processes were simplified. The most important part of the fiber analysis was the exact observance of the time periods given for boiling, in addition to the weighing procedures. 

After treatment with the obtained solution, the next step consisted of drying the insoluble residue, weighing it, and then calcinating it.

The acid detergent fiber (ADF) content represents the insoluble part of the sample that is left after boiling in acid detergent solution, from which the ash obtained upon calcination is subtracted:

(2) $$\%\;ADF=\frac{\left(\left(\chi -\alpha \right)-\left(\delta -\zeta \right)\right)\;\times \;100}{\beta },$$

(3) Blank value (ζ)=γ−ψ,

where

*α* = mass of FibreBag (g);
*β* = sample mass (g);
*χ* = mass of crucible and dried FibreBag, after digestion (g);
*δ* = mass of crucible and and ash (g);
*ζ* = blank value of empty FibreBag (g);
*γ* = mass of crucible and ash of the empty FibreBag (g);
ψ*=* mass of crucible (g).

### 2.7. Mineral Content Analysis of Studied Flours (S. latissima and Wheat Flour)

The content of potassium (K), calcium (Ca), magnesium (Mg), sodium (Na), copper (Cu), selenium (Se), manganese (Mn), chromium (Cr), molybdenum (Mo), iron (Fe), zinc (Zn), phosphorus (P), and iodine (I) from the marine algae *S. latissima*, was determined using the ICP-OES method (inductively coupled plasma—optical emission spectrometry). The sample was homogenized very well, and 0.250 g ± 0.025 g of the product was weighed in the mineralization vessel. Then, 1 mL of ultrapure water and 5 mL of concentrated HNO_3_ were added and left to pre-react for 15–30 min. The dishes were placed in the centrifuge and subsequently placed in the microwave oven. The determination was carried out at wavelengths specific to the target analytes. The detector signal for the specific wavelength was directly proportional to the concentration of the element in the solution.

A calibration curve was drawn from the average of the readings obtained with the reference solutions by graphically representing the average as a function of concentration (five concentration levels and a control—for K, Ca, Mg, Cu, Se, Mn, Cr, Mo, Fe, and Zn and four concentration levels and a control for P and I).

### 2.8. Total Polyphenol Content (TPC) of Studied Flours (S. latissima and Wheat Flour)

The total content of polyphenolic compounds was determined by the Folin-Ciocalteau (FC) method [25]. Methanol and water, in an equimolar ratio, were used as the extraction solvent. After vortexing (one hour at 10000 rpm) and centrifugation (one hour at 8000 rpm and temperature 20 °C), 1 mL extract was homogenized with 5 mL of FC reagent. After 5 min of incubation, 4 mL of sodium bicarbonate (7.5%) was added. The resulting mixture was kept in the dark for 20 min at room temperature to develop the color. The absorbance of the formed product was spectrophotometrically measured in VIS at a wavelength of 752 nm, using gallic acid as a standard. The total amount of polyphenols was expressed as gallic acid milliequivalents (GAE) per 100 g of fresh product.

### 2.9. Antioxidant Activity of Studied Flours (S. latissima and Wheat Flour)

The DPPH method, as described by Shekhar et al. [26], is used worldwide in terms of free radical scavenging quantification. The method is based on the color change from purple to yellow, with the color change occurring when the electron of the nitrogen atom is reduced by accepting a hydrogen atom from the antioxidant compounds of the stable organic radical 2,2-diphenyl-1-picrylhydrazyl.

The extraction solvent used is methanol and water in a stoichiometric ratio. To determine the antioxidant activity, 1 mL extract was homogenized with 6 mL of the DPPH methanolic solution. The mixture was left in the dark at a temperature of 20 °C for 30 min, and the absorbance was read at a wavelength of 517 nm. The antioxidant activity was expressed in Trolox milliequivalents per 100 g of fresh product.

### 2.10. Rheological and Enzymatic Properties 

The rheological and enzymatic behavior of the dough were determined using the Mixolab equipment, following the “Chopin +” protocol, according to ICC No. 173 [27] and as described by Oprea et al. [28]. The studied parameters are water absorption (%), stability (min), amplitude (Nm), α—strength of the protein chain (Nm/min), β—starch gelatinization rate (Nm/min), γ enzymatic downgrade speed (Nm/min), C1–C5—characteristic points on Mixolab curve, and TC1–TC5—specific time for C1–C5 points [28].

### 2.11. Bread Making

The experimental bread samples used in the study were obtained using the direct method and a standard recipe, as described by Oprea et al. [28]. The method of obtaining bread samples is based on using the same raw materials and the same protocol, consisting of mixing, fermenting time, fermenting, baking temperature, etc., for all bread samples that are further analyzed. 

Table 2 shows the technological parameters and the ingredients used to obtain the control sample, M, by the direct method. Samples A1, A2, A3, and A4 were made by substituting the total quantity of WF in the M sample with mixtures of WF and *S. latissima*, as shown in Table 1. The quantity of water used for each flour mixture was calculated by taking into consideration the values of the parameter CH (water absorbtion) determined by the Mixolab equipment, as described in Section 2.10.

### 2.12. Physicochemical Characteristics of the Experimental Bread

The rape seed displacement method was used in order to determine the specific volume of the bread (unit: cm^3^/g), in triplicate, according to SR 91:2007, AACC 2000 [29,30]. When evaluating porosity, the total scale of holes in a certain crumb volume was taken into account, together with the mass and density.

The crumb elasticity is represented in percentage and refers to the difference between the original height of the cylinder-shaped crumb bread and the height obtained by pressing and returning. Measurements of the moisture content entail drying 5 g of breadcrumbs to a constant weight at 103 °C (±2 °C). The reported data consist of the mean of triplicate measurements, each taken on a new loaf of bread. Titration of a bread extract, with 0.1 N NaOH solution in the presence of phenolphthalein as the indicator, was used to evaluate the acidity in degrees [29,30].

### 2.13. Sensory Analysis

The sensorial analysis of the bread samples was completed through two methods: a point-based method and a hedonic scale method. The hedonic scale method offers a general evaluation of the sensory characteristics of the sample, while the point-based method allows the identification of specific, deficient characteristics, at the level of which it is possible to intervene.

For the point-based method, a group of 10 panelists, both male and female, with ages ranging from 25 to 60, were selected, trained in accordance with ISO 8586:2012 [31], and evaluated all five bread samples. The panelists gave grades from 1 (lowest intensity) to 5 (highest intensity) for the following sensory attributes: crust and crumb color, crumb pore uniformity, crumb softness, bitter, salty and sour taste, specific flavor, and aftertaste. 

For the hedonic scale method, the consumer acceptability was determined on a 9-point hedonic scale, with 60 untrained panelists aged between 18 and 65 (60% females and 40% males). The panelists were chosen based on good health condition [32,33]. All bread samples of flour mixtures A1–A4 were compared to control samples, M.

### 2.14. Microbiological Analysis for Shelf Life

To establish the shelf life, the bread samples were subjected to microbiological analyses according to Order no. 27 of 2011 of ANSVA for matrices of simple bakery products for a period of four days. The determined parameters were yeasts, molds, and water activity [34].

### 2.15. Texture Analysis

The analysis of texture is based on the textural properties of breads measured using the Instron Texture Analyzer (model 5944, Illinois Tool Works Inc., Norwood, MA, USA), which has a 12 mm diameter compression piston. The testing was completed at room temperature and for a period of three days. The test parameters were compression speed at 100 mm/min, sample deformation at 40%, and load cell at 50 N. The thickness of the sample was approximately 20 mm (one slice). The testing was performed by compressing the sample twice.

Using the Bluehill 3.13 program, four texture parameters were calculated:

- firmness (hardness), (N);
- elasticity;
- cohesiveness;
- gumminess, (N).

### 2.16. Statistical Analysis

The mean values and standard deviations of the measured parameters were connected, and each analysis was carried out in triplicate. An analysis of variance (ANOVA) was performed, and Tukey’s test was used for the determination of significant differences between means. Calculations were performed assuming a significance level of α = 0.05.

## 3. Results and Discussions

### 3.1. Chemical Composition Analysis

The chemical characteristics of the *S. latissima* algae flour and the WF used in the study are presented in Table 3. Due to the high fiber and mineral content of *S. latissima*, its nutritional profile suggests that it could be used to fortify baked products. It is evident that the protein, fiber, potassium, magnesium, calcium, iron, phosphorus, and iodine contents of *S. latissima* are much higher than those of WF, except for the lower levels of fat, zinc, copper, selenium, and manganese.

From the presented values in Table 3, it can be seen that of the total constituent substances of seaweed flour, the ash content makes up 38.92%, indicating that the flour has a very high mineral content. Comparing with the data for crude protein, *S. latissima* has a value of 14.34% dry matter (DM), which is higher than that of wheat flour (11.37% DM). Also, the raw fiber content that represents a part of the total fiber (according to the method used to determine the crude fibers of which cellulose, hemicelluloses, and lignin are part) is above 6%. A crude fiber content of more than 6%, as part of the total fiber content, allows the nutritional claim of “rich in fiber”. Thus, regarding health claims, the claim regarding the increase in the volume of the fecal bowel is valid, as written in EU Regulation 432/2012 [35]. 

Seaweed is also important for its low Na/K ratio (0.24 for *S. latissima*, as can be seen in Table 3). This is significantly lower than for ingredients included in a variety of food products, such as sausages (4.9), cheddar cheese (8.7), and olives (43.6) [36,37]. Similar to Na and K, the consumption of Ca and Mg is associated with cardiovascular health.

Because magnesium acts as a calcium antagonist on smooth muscle tone, it relaxes blood vessels, which may reduce blood pressure when taken as directed [38]. Since an insufficient magnesium intake can lead to an excessive calcium buildup in soft tissues, which can induce kidney stones and arthritis, the Ca/Mg ratio is also crucial for calcium absorption [39,40]. 

The trace elements iron (Fe), manganese (Mn), copper (Cu), zinc (Zn), molybdenum (Mo), selenium (Se), and iodine (I), which are necessary for maintaining good health, can also be found in seaweeds, in addition to the aforementioned minerals [41,42,43]. 

Iodine is necessary for thyroid hormone production and is, therefore, essential for healthy growth and development [44]. High quantities of Se were also discovered by Netten et al. [45] in brown algae from Japan, mainly in *S. latissima* (6 mg/kg DW). According to Schiener et al. [46] and Küpper et al. [47], *Laminaria* spp. and *S. latissima* were discovered to have significant amounts of iodine. *Laminariales* have a special propensity for iodine accumulation, primarily as iodide and sporadically as iodate. Iodine concentrations significantly differ between species and due to the specific conditions of growth (harvest conditions, seasonality, and depth), among other things [43,46]. It was shown that Japanese people, who are thought to have iodine-sufficient diets, consume large amounts of seaweed, resulting in concentrations of iodine above the upper limit of tolerated intake (600 µg for adults). This caused a significant change in the study participants’ serum levels of the thyroid hormones, such as free thyroxine, free triiodothyronine, and serum thyrotropin. However, the values quickly recovered to normal as the individuals resumed their regular eating habits [48].

It is significant to remember that among populations considered to be iodine-deficient, slight increases in iodine consumption may also change the pattern of thyroid illnesses. This happens as a result of the thyroid accumulating a higher percentage of ingested iodine at low dietary doses, more efficiently utilizing iodine from the breakdown of thyroid hormones and decreasing the renal excretion of iodine. [49]. Because of this, care must be taken while consuming iodine-rich seaweed. Despite growing public acceptability, seaweed is still seen as an exotic cuisine in Europe, and consumption should not exceed the advised amounts [50].

Analyzing data from Table 3 for Ca, Mg, K, Fe, P, and I levels, *S. latissima* has a calcium content of 8236 mg/kg. This is 18.6 times greater than the calcium content of WF (441 mg/kg). *S. latissima* has a magnesium level of 6041 mg/kg, which is 12.59 times greater than the value of 479.7 mg/kg found in WF. The amount of K in *S. latissima* is 62,088 mg/kg, which is 33.07 times more than the amount of K found in WF (1877 mg/kg). The Fe content for *S. latissima* is 35.23 mg/kg, a value almost double that of WF (18.37 mg/kg). Regarding the P content, *S. latissima* has a value of 2263 mg/kg, approximately 2.91 times higher than for WF (776 mg/kg). The I content for *S. latissima* reaches a record level of 1253 mg/kg.

A lot of research has asserted that because seaweeds are rich in minerals, the consumption of these significantly help people reaching their recommended daily intakes (RDI) of certain important minerals that typically are lacking in diets [51,52,53]. Considering the fact that seaweeds have a high Na content, their use should be limited in a Western European diet already overloaded with this element. 

The use of algae as an ingredient gives the advantages of incorporating other minerals like calcium, potassium, and iodine, which typically are missing or at low levels in conventional diets. It also gives the benefit of reducing the amount of salt in standardized recipes (Table 3). The TPC value of 283.5 mg GAE/100 g and the antioxidant value (13.10 mg Trolox/100 g) of the *S. latissima* flour (presented in Table 3) highlight the nutritional importance of the *S. latissima* algae used in this study. It is important to keep in mind when comparing TPC research with other scientific studies that an exhaustive extraction method is nearly impossible to develop because of the polyphenol class’s extreme chemical complexity. Furthermore, when these compounds naturally form complexes with other molecules like proteins, polysaccharides, and lipids, the extraction process becomes more difficult. It is widely acknowledged, nevertheless, that polyphenols are easily separated from one another by polar solvents, such as alcohols like methanol and ethanol and ketones like acetone, because of their phenolic (hydrophilic) nature [54]. 

For instance, the dried G. changii harvested in the mangrove area of Santubong (Malaysia) had TPCs equal to 1083 mg GAE/100 g, 858 mg GAE/100 g, and 820 mg GAE/100 g, respectively, in ethanol, methanol, and acetone extracts. G. vermiculophylla from the coastal areas of Denmark showed TPCs of 51.4 mg GAE/100 g and 95.2 mg GAE/100 g on a dw basis, respectively, in water and ethanol extracts, whereas the methanol extract of G. edulis collected in Tamil Nadu (India) reported a total amount of phenolic compounds equal to 32,700 mg GAE/100 g [54]. 

G. gracilis coming from the west coast of Ireland showed TPC data lower than those reported in other studies, as aqueous methanol and aqueous ethanol extracts were, respectively, characterized by TPCs equal to 536 mg GAE/100 g and 476 mg GAE/100 g [54].

### 3.2. Rheological Properties of Doughs Obtained from Flour Mixtures

Table 4 displays the outcomes of the Mixolab device’s analysis of the flour mixtures’ rheological characteristics, compared to the control sample, M, of WF, using the rheological parameters that were found in the Mixolab Chopin+ protocol for (ICC Standard 173) [27]. 

The measurements made for sample M (type 650 WF) revealed rheological behavior particular to baking flour with medium technological characteristics: water absorption (CH) = 58.1%; stability (ST) = 8.78 (min); amplitude = 0.091 (Nm); and TC1 dough development time = 1.2 min. Similar findings were made by Mironeasa et al. [55] and Apostol et al. [56], who recorded values of 61% for water absorption (CH), 8.9 min for stability (ST), and 1.68 min for TC1. The parameters that characterize the rheological and enzymatic behavior are α = −0.080 (Nm/min) (strength of the protein chain); β = 0.112 (Nm/min) (starch gelatinization rate); γ = −0.024 (Nm/min) (enzymatic downgrade rate); C2 = 0.417 (Nm), TC2 = 17.37 min; C3 = 1.793 (Nm), TC3 = 27.95 min; C4 = 1.740 (Nm), TC4 = 30.82 min; and C5 = 2.731 (Nm), TC5 = 45.00 min, which shows a normal behavior from the point of view of the bakery standard. Several other studies [57,58] achieved comparable results by utilizing similar raw materials and analysis tools. Comparatively, looking at the values reported in Table 4, it is evident that the water absorption capacity (CH) increased along with the proportion of *S. latissima* flour, i.e., 58.1% (M), 59.0% (A1), 60.8% (A2), 62.7% (A3), and 62.6% (A4). This gives a maximum increase of 7.91% from M to A3, a fact that can be explained by the high fiber content. 

Additionally, it should be highlighted that the maximum value of A4 falls at the upper end of the 55–62% range that is ideal for the production of baked goods [59], which confirms that the highest amount of *S. latissima* flour was correctly chosen. Similar results for enhanced water absorption were observed by Hasmadi et al. [60].

The dough stability increased from 8.78 min (control sample, M) to 10.33 min (A1), 10.37 min (A2), 10.83 min (A3), and 11.83 min (A4), corresponding to a maximum rise from M to A4 of 34.73% (Table 4). This implies that these flour mixtures slightly exceed the optimal limit for their inclusion in flours with optimal stability for breadmaking [59]. There were no statistically significant variations in the amplitude. Similar outcomes were also attained by Hasmadi et al. [60], who, for an 8% red seaweed (*Kappaphycus alvarezii*) powder addition, obtained a stability value of 11.90. Analysis of the rheological and enzymatic reactions that follow the stabilization period of the dough revealed considerable variations (Table 5) brought on by the addition of *S. latissima*.

The strength of the protein chain was expressed by the values of the α slope, which increased from 0.080 Nm/min (M) to 0.098 Nm/min for A1 and 0.120 Nm/min for A2 but then decreased to 0.098 Nm/min for A3 and 0.044 Nm/min for A4. This suggests that when the amount of *S. latissima* flour increases, the protein chains more quickly degrade as a result of mechanical and heat action. However, for A4, the effect of degradation is lower than that of the control sample, M, which is probably due to the interaction between the wheat gluten network and seaweed particles.

The β slope values, which represent the starch gelling phenomenon, increased from 0.112 Nm/min (M) to 0.374 Nm/min for A1, 0.418 Nm/min for A2, 0.424 Nm/min for A3, and 0.436 Nm/min for A4, an increase from of 289.28% M to A4 (Table 4). The dough consistency decreased from C3 = 1.793 Nm (M) to C3 = 1.576 Nm (A3), a decrease of 13.76% from M to A3 (Table 4). 

In the third phase, when the temperature reaches 50–55 °C, the starch gel is formed. At this point, amylose molecules are produced and have the ability to bind water, increasing the viscosity, as starch granules swell and absorb water. The lowest C3 was achieved in the case of the dough formed from the mixture of pure wheat flour with 4.5% algae flour A3) (Table 4). 

**Figure 1:**
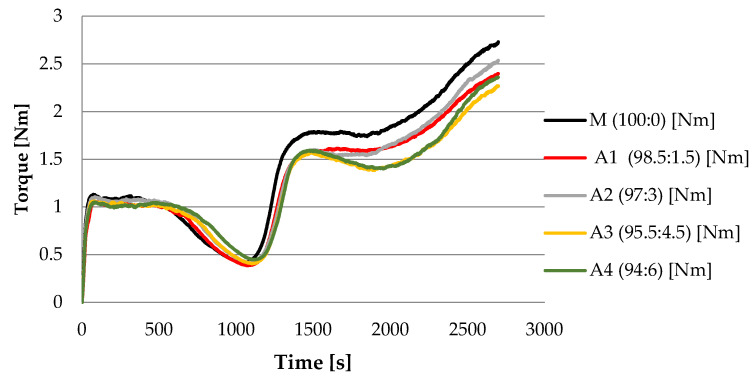
The effect of *S. latissima* flour substitution level on Mixolab curves for the torque.

As the percentage of algal flour increases, starch gelatinization is disturbed. The maximal consistency point, C3, is achieved faster, indicating a substantially quicker gelatinization process (a feature also evidenced by the increase in β slope values), as shown in Figure 1, based on an analysis of the progression of TC3 values in Table 4.

Regarding the γ parameter, we observe that the absolute values for the A3 and A4 samples are higher than for the M, A1, and A2 samples. This shows a higher enzymatic activity for A3 and A4 compared to samples with less or no seaweed flour. The stability of the hot formed starch gel, expressed by the parameter C4, gradually decreases as the percentage of algae flour increases. The stability time of the formed gel (TC4) decreases at A1 and A2 and then increases at A3 and A4 compared to the control sample. During the cooling stage of the dough, the starch retrogradation phase takes place (C5), a phenomenon that leads to an increase in the consistency of the dough. When analyzing WF and mixtures of WF with *S. latissima*, a decrease in C5 values was observed with similar differences in starch gel stability.

### 3.3. Bread Quality

The SR 91/2007 [29] standard was followed by analytical procedures of the quality attributes for the bread samples. The goal was to identify the physicochemical indicators required for the assessment of the bread quality, as presented in Table 5. From the mixtures of WF and *S. latissima*, four bread samples were obtained and examined in comparison to the control sample, M. The bread samples were coded in the same manner as the flour mixtures shown in Table 1. The physical and chemical indicators for each of the samples under study are shown in Table 5, with values that fall within the parameters of SR 878/1996 [61] and with a minimum porosity value of 66% and a maximum acidity value of 3.5 degrees [61].

We observe that as the content of seaweed flour increases, the moisture increases from 44.84 (M) to 45.98 (A4), due to the different values of the degree of water absorption during dough formation and water losses from the baking time. However, the addition of algal flour has a moderate effect on the moisture content of the samples. The volume of the loaves decreased from 372 cm^3^ (M) to 285 cm^3^ (A4), due to certain changes in the structure of the gluten network. However, the sample volume falls within the limits provided by the SR 878/96 standard for white bread (min. 280 cm^3^). The porosity of the samples falls within the normal limits of white bread (min. 74%), provided in SR 878/96 [61].

The control sample, M, and the samples made from the flour mixtures have the same values for elasticity. As for the acidity of the samples, this corresponds to the rules for checking the quality of white bread (max. 3.5 degree acidity) of the same standard. A slight decrease in the acidity of the two bread samples A3 and A4 is observed. The outcomes of the performed studies of the physicochemical properties of bread samples containing *S. latissima* powder were consistent with the findings of the rheological and enzymatic analyses of the flour combination samples. Thus, samples A1 (mixture of 98.5% WF + 1.5% *S. latissima*) and A2 (mixture of 97% WF + 3% *S. latissima*) demonstrate permissible changes in the rheological parameter values for acceptable technological behavior and bread product quality. The rheological behavior is seen to be negatively affected in the case of the other samples with higher amounts of *S. latissima* powder.

**Figure 2:**
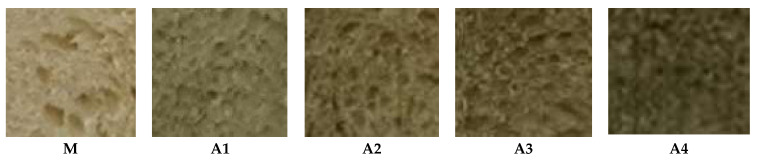
Bread samples (A1–A4) comparatively presented to the test sample (M).

As can be seen from Figure 2, as the percentage of algal flour increases, the color of the bread samples darkens compared to the color of the control sample (M). Also, the volume and the porosity are depreciated as the percentage of algal flour increases.

### 3.4. Sensory Evaluation

One day after processing, the bread samples underwent sensory evaluation to ensure that they were in comparable consumption conditions to the standard bread assortments in the bakery sales network. In order to create a product with functional potential, experimental samples of bread with the addition of *S. latissima* powder were compared to the control sample (M) and to one another. The goal was to identify the ideal percentage of acceptable ingredients from a sensory point of view. Equal importance factors were formed for each of the 10 features identified during the sensory assessment of the bread samples, and the arithmetic mean of the scores for each sample was utilized to interpret the findings. As part of the process of creating a new bread product, a comparative analysis was conducted, leading to the selection of this streamlined calculation approach. Table 6 lists the sensory characteristics and scores for each characteristic that were used on the sensory assessment form.

**Figure 3:**
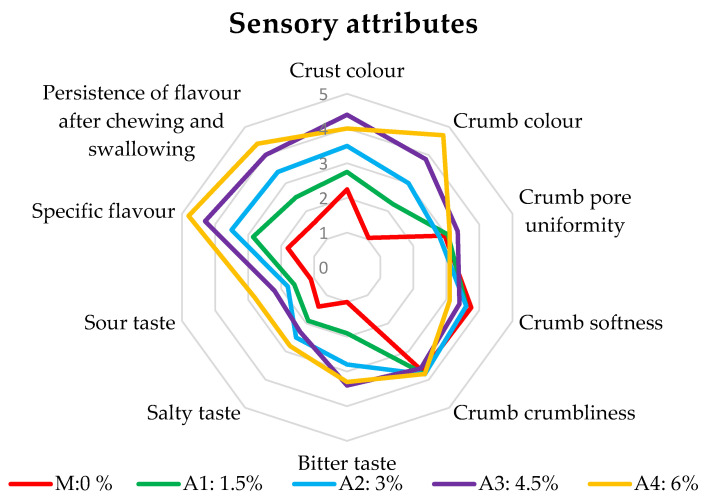
Sensory attributes obtained by panel analysis (point scale method).

As shown in Table 6, the color of the crumb of the samples darkened as more *S. latissima* flour was added. Accordingly, the average rating provided by the judges increased from 2.25 (sample M) to 4 (A4). The average score given by the judges for crumb softness was 3.75 for the M sample and decreased to a minimum of 3.10 for the A4 sample, which supports the idea that the quality of the samples declined as the amount of *S. latissima* flour increased. The values of the taste and flavor characteristics in Table 6 show that when the amount of *S. latissima* is increased, the bitter, sour, and salty flavors, as well as a particular flavor, significantly grew. After chewing and swallowing, the flavor’s persistence significantly increased, rising from 1.60 for M to 4.40 for A4, with all values of the A1–A4 samples higher than the value for the control sample, M. The cumulative sensory analysis of the examined bread samples is shown in Figure 3. With marks ranging from 0 to 5, each axis represents a sensory analysis criterion (sour taste, salty taste, etc.). A certain contour with a particular color is produced by joining the dots that correspond to the marks given by panelists for a specific test (Table 6). The way the outlines overlap reveals how samples A1–A4 are enclosed in comparison to the control sample, M, which the consumer is used to. It can be seen that for sample A1 (green color), almost all the characteristics were similar to the control sample, M (red color). 

From the analysis of the sensory evaluation by the panelists, it appears that the average of the sensory attributes of the crumb crumbliness, the crumb softness, and the crumb pore uniformity is equal or very close to the sensory attributes of all the experimental samples (Figure 3). From the point of view of the other attributes, as the percentage of algal flour increased, the corresponding values increased in an unfavorable direction compared to the control sample. In particular, this was the case for the specific flavor and the persistence of the flavor after chewing and swallowing. 

Samples A3 (purple color) and A4 (orange color) (bread obtained from the mixture of wheat flour with 4.5% algae flour and 6% algae flour) scored much less favorably than the control sample M (Figure 3).

**Figure 4:**
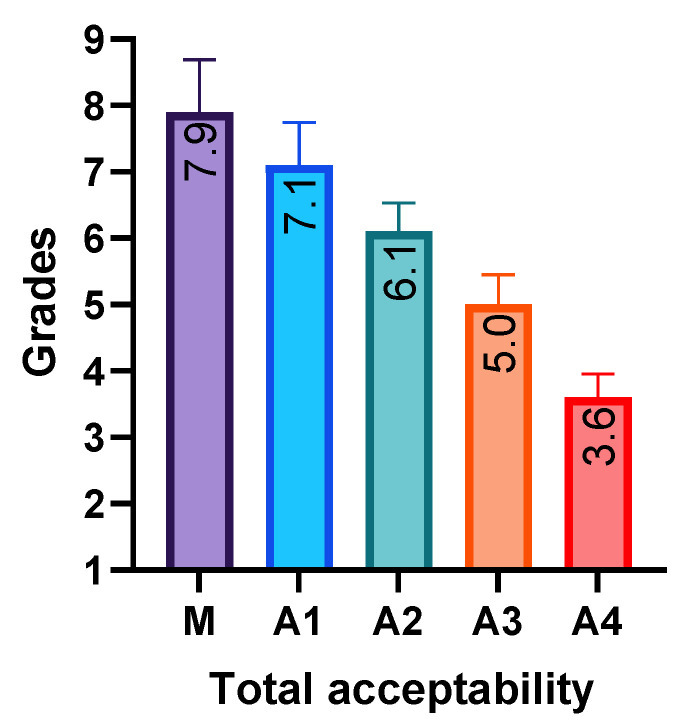
Sensory analysis results obtained by analyzing acceptance tests of the various bread samples (hedonic scale method).

The acceptance values obtained for the experimental samples with the addition of seaweed flour are presented in Figure 4. The acceptance tests applied the nine-point hedonic scale, with nine possible qualifications to be given to the evaluated product: 9—I like it extremely; 8—I like it very much; 7—I like it moderately; 6—I like it slightly; 5—indifferent; 4—I dislike it slightly; 3—I dislike it moderately; 2—I dislike it very much; 1—I dislike it extremely. The graph shown in Figure 4 was created based on the provided grades and shows that the samples with the addition of 1.5% and 3% *S. latissima* flour received higher ratings than the samples with 4.5% and 6% *S. latissima* flour.

From the results of the acceptance tests, the control sample, M, received a score of 7.9, i.e., between *I* like it very much and I like it moderately. For the samples with *S. latissima* flour mixtures, A1 scored 7.10, i.e., between I like it very much and I like it moderately; A2 scored 6.10, i.e., between I like it moderately and I like it slightly; A3 scored 5.00, meaning indifferent; and A4 obtained a score of 3.60, i.e., between I dislike slightly and I dislike moderately. It can be concluded that sample A1 has sensory attributes close to the control sample and that sample A2 obtained an acceptable score compared to the control sample, M.

### 3.5. Shelf Life Estimation Based on Microbiological Activity

To establish the validity period, the bread samples were subjected to microbiological analyses according to Order no. 27 of 2011 of ANSVA for matrices of simple bakery products [34]. The determined parameters were yeasts and molds and water activity. The results of the microbiological analyses of bread samples made from wheat flour and with the addition of algae flour are presented in Table 7.

It can be concluded that the samples of bread with the addition of algae fall within the limits of 100 cfu/g, as stipulated in SR 27:2011 [34], from a microbiological point of view, for at least 96 h when stored at room temperature and packed in a paper bag. As for the obtained values for the water activity during storage, the parameter slightly decreased in all samples. The optimal water activity value, according to Annex A (informative) of the SR ISO 21527-2:2009 [62] standard, must be ≥0.95 for bread products.

### 3.6. Texture Analysis

Instrumental texture measurement compresses the sample by exerting pressure while measuring the product-specific texture parameters. The firmness represents the maximum value of the force required for the first compression of the sample and sensorially correlates with the softness of the crumb. A high firmness means a denser, harder crumb. Table 8 shows that sample A4 has the highest firmness during the 3 days of analysis (2.14 N Day 1; 3.3 N Day 2; 3.67 N Day 3), followed by sample A3, A2, A1, and the control sample, M (1.14 N Day 1; 2.03 N Day 2; 2.21 N Day 3). Samples M and A1 have the lowest firmness, i.e., the highest softness.

Elasticity represents the ability of the bread crumb to return to its original shape after being deformed during a first compression. From Table 8, we note that sample A4 recorded the highest elasticity on the first and second days of analysis (0.99 and 1, respectively). This implies that the sample returns to its initial form the fastest, though unlike on Day 3, for which the sample recorded the lowest elasticity (0.97). Samples M, A1, and A2 turned out to have the same elasticity value throughout the 3 days of analysis, 0.98.

Cohesiveness represents the resistance of the sample to a secondary deformation compared to the reaction of the sample to the first deformation. Table 8 shows the cohesiveness of the samples, which is approximately the same in all samples between 0.73 (A3 and A4) and 0.78 (A2). Cohesiveness decreases during the 3 days of analysis, which means that at the second deformation the samples offer less resistance.

Gumminess is the energy required to disintegrate a semi-solid product into a good-to-swallow shape and correlates with firmness. In Table 8, we observe that sample A4 has the highest gumminess (1.55 N Day 1; 2.12 N Day 2; 1.98 N Day 3). Sample A4 also has the highest firmness. For gumminess, sample A1 (0.94 N Day 1; 1.53 N Day 2; 0.95 on Day 3) is closest to the control sample M (0.89 N Day 1; 1.23 N Day 2; 1.25 N Day 3). The fact that all bread samples were made using just the raw materials mentioned in Table 2, without any preservatives, led to high firmness values and reduced the period of data collection to three days and not four, like in the case of the shelf life analysis.

Thus, the addition of algae flour caused an increase in the firmness and gumminess of the samples, from M to A4. Meanwhile, elasticity did not show significant changes across the samples. Cohesiveness slightly decreased with the addition of algae, and an important change in cohesiveness was observed between Day 1 and Day 2, though less than that between Day 2 and Day 3. The gumminess of the samples increased with the addition of algae and mostly increased between Day 1 and Day 2.

## 4. Conclusions

The addition of *S. latissima* flour in standard bakery recipes brings high-level quantities of minerals, such as K, Fe, Ca, P, and I, as well as an important amount of fiber, and, thus, enhances the crumb matrix’s nutritional quality of wheat flour. For the manufacturing of bread goods, replacing more and more wheat flour with *S. latissima* flour (beyond 3%) results in deteriorating rheological parameters and poorer technological performance. 

The baked goods produced under these circumstances have a limited volume and a compact texture. The sensory examinations of the panelists concluded that sample A1 (1.5% algae flour) was close to many of the characteristics of the control sample, M (WF type 650). For sample A2 (3% algae flour), the panelists concluded that with respect to porosity, crumb crumbliness, and crumb softness, the characteristics of the sample were close to those of the control sample M.

Regarding the crumb color and crumbliness, samples A3 (4.5% algae flour) and A4 (6% algae flour) exceeded the boundaries of the control sample. Sour, salty, and bitter tastes and a specific flavor were significant for both the A3 and A4 samples with the *S. latissima* addition.

Shelf life analysis performed for 96 h indicates microbiological activity and water activity that falls within the limits of the regulation in force. Textural analyses of the bread samples show an increase in firmness and gumminess over time and with the increase in algae addition. No significant changes regarding elasticity are observed. In terms of cohesiveness, the addition of algae does not produce significant changes, but all samples show up to a 30% decrease over time. The results obtained in this study demonstrate the potential of using S. latissima flour in proportions of a maximum of 3% as a functional ingredient in the production of bakery products with a high nutritional value. 

## Figures and Tables

**Table 1:** Coding of experimental flour samples.

Samples	Sample Composition (*w*/*w*)
M	Sample 0—100% wheat flour, type 650
A1	Sample 1—98.5% WF, type 650 + 1.5% dried *S. latissima* flour
A2	Sample 2—97% WF, type 650 + 3% dried *S. latissima* flour
A3	Sample 3—95.5% WF, type 650 + 4.5% dried *S. latissima* flour
A4	Sample 4—94% WF, type 650 + 6% dried *S. latissima* flour

**Table 2:** Technological parameters and the ingredients used to obtain the control sample, M.

Control Sample (M)
Direct Method Recipe
Wheat flour type 650: 1 kg
Yeast (Pakmaya): 0.030 kg
Salt: 0.015 kg
Water: ≈ 0.750 L
Kneading: 2 min at 60 rpm followed by 8 min of 90 rpm in the mixer. Fermentation 1: 90 min at 30 °C, 70% relative humidity. Splitting into equal parts. Modeling into round shapes; second fermentation for 10 min; modeling into final shapes. Fermentation 2: 40 min at 30 °C, 70% relative humidity. Baking for 40 min at 240 °C, steaming the samples in the first 10 s. Cooling to 21 °C for 2 h.

**Table 3:** Chemical composition of *S. latissima* seaweed and wheat flour.

Parameter	*S. latissima* Flour	CV (%)	Wheat Flour (WF) 650	CV (%)	*p*-Value (*t*-Test)
Moisture content (%)	7.170 ± 0.22 ^a^	1.534	13.1 ± 0.02 ^b^	0.076	<0.0001
Ash (%)	38.92 ± 4.25 ^a^	5.515	0.660 ± 0.05 ^b^	4.009	<0.0001
Protein (%)	14.34 ± 1.02 ^a^	3.825	11.37 ± 0.30 ^b^	1.366	0.0008
Fat (%)	0.706 ± 0.03 ^a^	2.162	1.167 ± 0.10 ^b^	4.949	0.0002
Raw fiber (%)	6.367 ± 0.46 ^a^	3.614	1.233 ± 0.05 ^b^	2.040	<0.0001
Potassium (mg/kg DM)	62,088 ± 1949 ^a^	1.577	1877 ± 15.80 ^b^	0.420	<0.0001
Magnesium (mg/kg DM)	6041 ± 166.0 ^a^	1.424	479.7 ± 10.41 ^b^	1.088	<0.0001
Calcium (mg/kg DM)	8236 ± 636.0 ^a^	3.881	441.8 ± 7.810 ^b^	0.893	<0.0001
Iron (mg/kg DM)	35.23 ± 6.20 ^a^	8.823	18.37 ± 1.610 ^b^	4.395	<0.0001
Na (mg/kg DM)	15,205 ± 35.00 ^a^	0.117	20.98 ± 1.880 ^b^	4.483	<0.0001
Zinc (mg/kg DM)	30.06 ± 0.33 ^a^	0.572	55.40 ± 2.50 ^b^	2.305	<0.0001
Copper (mg/kg DM)	0.846 ± 0.03 ^a^	1.804	1.197 ± 0.030 ^b^	1.276	<0.0001
Selenium (mg/kg DM)	0 ^a^	0	0.041 ± 0.006 ^b^	7.391	<0.0001
Manganese (mg/kg DM)	3.967 ± 0.50 ^a^	6.344	5.183 ± 1.490 ^a^	14.370	0.0552
Chromium (mg/kg DM)	0 ^a^	0	0.079 ± 0.012 ^b^	7.595	<0.0001
Molybdenum (mg/kg DM)	0 ^a^	0	0.1321 ± 0.002 ^b^	1.024	<0.0001
Phosphorus (mg/kg d.m.)	2263 ± 129.0 ^a^	3.032	776.0 ± 88.00 ^b^	5.710	<0.0001
Iodine (mg/kg DM)	12,530 ± 2076 ^a^	8.307	0.1 ± 0.020 ^b^	10.00	<0.0001
Total polyphenols (mg GAE/100 g)	283.5 ± 13.79 ^a^	2.643	0 ^b^	0	<0.0001
DPPH (mg Trolox/100 g; µmol T/100 g)	13.10 ± 0.20 ^a^	0.076	0 ^b^	0	<0.0001

Note: Different letters in the same row indicate significant differences (*p* < 0.05); CV, coefficient of variation.

**Table 4:** Rheological and enzymatical characteristics of doughs obtained from WF and *S. latissima* (S.l.) flour mixtures.

Parameter	M (100% WF)	A1 (98.5% WF + 1.5% S.l.)	A2 (97% WF + 3% S.l.)	A3 (95.5% WF + 4.5% S.l.)	A4 (94% WF + 6% S.l.)
Water absorption (%)	58.1 ± 0.05 ^a^	59.0 ± 0.06 ^b^	60.80 ± 0.05 ^c^	62.70 ± 0.01 ^d^	62.60 ± 0.01 ^d^
Stability (min)	8.78 ± 0.28 ^a^	10.33 ± 0.25 ^b^	10.37 ± 0.17 ^b^	10.83 ± 0.14 ^c^	11.83 ± 0.1 ^d^
Amplitude (Nm)	0.091 ± 0.01 ^a^	0.099 ± 0.01 ^b^	0.112 ± 0.01 ^c^	0.102 ± 0.01 ^b^	0.089 ± 0.01 ^a^
Moisture (%)	11.90 ± 0.5 ^a^	11.90 ± 0.4 ^a^	12.50 ± 0.5 ^c^	12.50 ± 0.2 ^c^	12.20 ± 0.1 ^b^
α	−0.080 ± 0.002 ^a^	−0. 098 ± 0.003 ^b^	−0.120 ± 0.002 ^c^	−0.098 ± 0.003 ^b^	−0.044 ± 0.002 ^d^
β	0.112 ± 0.003 ^a^	0. 374 ± 0.003 ^b^	0.418 ± 0.004 ^c^	0.424 ± 0.003 ^d^	0.436 ± 0.004 ^e^
γ	−0.024 ± 0.003 ^b^	0.030 ± 0.002 ^d^	−0.010 ± 0.002 ^a^	−0.036 ± 0.006 ^c^	−0.026 ± 0.05 ^b^
C1	1.132 ± 0.01 ^a^	1.056 ± 0.03 ^c,d^	1.107 ± 0.03 ^b^	1.063 ± 0.04 ^c^	1.052 ± 0.02 ^d^
TC1	1.20 ± 0.1 ^a^	1.27 ± 0.08 ^a,b^	1.37 ± 0.06 ^c^	1.35 ± 0.07 ^c^	1.33 ± 0.05 ^b,c^
C2	0.417 ± 0.01 ^a^	0.417 ± 0.02 ^a^	0.380 ± 0.02 ^b^	0.406 ± 0.01 ^a^	0.448 ± 0.01 ^c^
TC2	17.37 ± 0.13 ^a^	17.93 ± 0.11 ^b^	17.93 ± 0.08 ^b^	18.37 ± 0.011 ^c^	18.65 ± 0.012 ^d^
C3	1.793 ± 0.02 ^a^	1.699 ± 0.02 ^b^	1.601 ± 0.01 ^c^	1.576 ± 0.01 ^d^	1.594 ± 0.02 ^c,d^
TC3	27.95 ± 0.32 ^a^	23.00 ± 0.45 ^d^	25.03 ± 0.28 ^b^	24.73 ± 0.51 ^b,c^	24.58 ± 0.29 ^c,d^
C4	1.740 ± 0.01 ^a^	1.596 ± 0.01 ^b^	1.534 ± 0.01 ^c^	1.401 ± 0.01 ^d^	1.385 ± 0.01 ^e^
TC4	30.82 ± 0.17 ^a^	30.60 ± 0.13 ^a^	30.48 ± 0.10 ^a^	31.62 ± 0.12 ^b^	32.57 ± 0.15 ^c^
C5	2.731 ± 0.08 ^a^	2.540 ± 0.10 ^b^	2.397 ± 0.09 ^c^	2.276 ± 0.10 ^d^	2.362 ± 0.11 ^c,d^
TC5	45.00 ± 0.01 ^a^	45 ± 0.01 ^a^	45.02 ± 0.01 ^a^	45.02 ± 0.01 ^a^	45.02 ± 0.01 ^a^

Note: Different letters in the same row indicate significant differences (*p* < 0.05).

**Table 5:** Physicochemical indicators of bread samples with the addition of *S. latissima*.

Sample	Mass (kg)	Specific Volume (cm^3^/100 g)	Porosity (%)	Elasticity (%)	Humidity (%)	Acidity (Degree)
P0: 0%	0.489 ± 0.01 ^a^	372 ± 4.46 ^a^	83 ± 1.66 ^a^	96 ± 0.96 ^a^	44.84 ± 0.90 ^a^	1.4 ± 0.04 ^a^
A1	0.491 ± 0.03 ^a^	363 ± 4.36 ^b^	82 ± 1.64 ^b^	96 ± 0.96 ^a^	45.53 ± 0.91 ^b^	1.4 ± 0.04 ^a^
A2	0.495 ± 0.02 ^a^	345 ± 4.14 ^c^	81.2 ± 1.62 ^c^	97 ± 0.97 ^b^	45.88 ± 0.92 ^c^	1.4 ± 0.03 ^a^
A3	0.498 ± 0.02 ^a^	311 ± 3.73 ^d^	77 ± 1.54 ^d^	96 ± 0.96 ^a^	45.93 ± 0.92 ^d^	1.2 ± 0.02 ^b^
A4	0.499 ± 0.02 ^a^	285 ± 3.42 ^e^	74.7 ± 1.49 ^e^	97 ± 0.97 ^b^	45.98 ± 0.92 ^e^	1.2 ± 0.01 ^b^

Note: Data with different letters in each column are significantly different for *p* < 0.05.

**Table 6:** Summary of scores obtained by the panel evaluation of samples for bread samples with the addition of *S. latissima*.

Sensorial Attribute	Crust Color	Crumb Color	Crumb Pore Uniformity	Crumb Softness	Crumb Crumbliness	Bitter Taste	Salty Taste	Sour Taste	Specific Flavor	Persistence of Flavor after Chewing and Swallowing
Samples										
M: 0%	2.25 ± 0.05 ^a^	1.05 ± 0.02 ^a^	2.95 ± 0.21 ^a^	3.75 ± 0.08 ^d^	3.70 ± 0.07 ^a^	1.00 ± 0.02 ^a^	1.40 ± 0.03 ^a^	1.10 ± 0.02 ^a^	1.80 ± 0.04 ^a^	1.60 ± 0.03 ^a^
A1: 1.5%	2.75 ± 0.06 ^b^	2.25 ± 0.05 ^b^	3.05 ± 0.27 ^a^	3.60 ± 0.07 ^c^	3.80 ± 0.08 ^b^	1.90 ± 0.04 ^b^	1.90 ± 0.04 ^b^	1.60 ± 0.03 ^b^	2.85 ± 0.06 ^b^	2.50 ± 0.05 ^b^
A2: 3%	3.5 ± 0.07 ^c^	3.00 ± 0.06 ^c^	2.80 ± 0.39 ^a^	3.60 ± 0.07 ^c^	3.80 ± 0.08 ^b^	2.80 ± 0.06 ^c^	2.50 ± 0.05 ^c^	1.80 ± 0.04 ^c^	3.50 ± 0.07 ^c^	3.40 ± 0.07 ^c^
A3: 4.5%	4.4 ± 0.09 ^e^	3.85 ± 0.08 ^d^	3.35 ± 0.07 ^a^	3.40 ± 0.07 ^b^	3.60 ± 0.25 ^a,b^	3.40 ± 0.07 ^d^	2.30 ± 0.05 ^b, c,d,e^	2.20 ± 0.04 ^d^	4.30 ± 0.09 ^d^	4.00 ± 0.08 ^d^
A4: 6%	4 ± 0.08 ^d^	4.7 ± 0.09 ^e^	3.10 ± 0.25 ^a^	3.10 ± 0.06 ^a^	3.80 ± 0.08 ^b^	3.30 ± 0.20 ^c,d,e^	2.80 ± 0.06 ^e^	2.80 ± 0.06 ^e^	4.80 ± 0.10 ^e^	4.40 ± 0.09 ^e^

Note: Data with different letters in each column are significantly different for *p* < 0.05.

**Table 7:** Microbiological indicators of bread samples with the addition of *S. latissima* flour.

Sample	Yeasts and Molds cfu/g	Water Activity Aw
Initial analysis		
M	<10	0.968 ± 0.019 ^a^
A1	<10	0.974 ± 0.019 ^b^
A2	<10	0.972 ± 0.019 ^c^
A3	<10	0.972 ± 0.019 ^c,d^
A4	<10	0.972 ± 0.019 ^c,d,e^
Analysis after 48 h		
M	<10	0.960 ± 0.019 ^a^
A1	<10	0.971 ± 0.019 ^b^
A2	<10	0.969 ± 0.019 ^c^
A3	<10	0.970 ± 0.019 ^d^
A4	<10	0.969 ± 0.019 ^c,e^
Analysis after 72 h		
M	3.0 × 10^1^	0.917 ± 0.018 ^a^
A1	4.0 × 10^1^	0.965 ± 0.019 ^b^
A2	4.0 × 10^1^	0.951 ± 0.019 ^c^
A3	3.0 × 10^1^	0.963 ± 0.019 ^d^
A4	7.0 × 10^1^	0.950 ± 0.019 ^e^
Analysis after 96 h		
M	6.0 × 10^1^	0.907 ± 0.018 ^a^
A1	6.0 × 10^1^	0.944 ± 0.019 ^b^
A2	6.0 × 10^1^	0.947 ± 0.019 ^c^
A3	7.0 × 10^1^	0.953 ± 0.019 ^d^
A4	9.0 × 10^1^	0.940 ± 0.019 ^e^

Note: Data with different letters in column/day are significantly different for *p* < 0.05.

**Table 8:** Textural analysis of bread samples with the addition of *S. latissima* flour.

Samples		M	A1	A2	A3	A4
Analysis						
Firmness (Force 40%) (N)	Day 1	1.14 ± 0.07 ^a^	1.3 ± 0.17 ^a, b^	1.37 ± 0.09 ^b^	1.95 ± 0.18 ^c,d^	2.14 ± 0.04 ^d^
	Day 2	2.03 ± 0.12 ^a^	2.52 ± 0.02 ^b^	2.51 ± 0.27 ^a,b^	2.92 ± 0.22 ^b, c^	3.30 ± 0.35 ^b,c,d^
	Day 3	2.21 ± 0.26 ^a^	1.87 ± 0.14 ^a^	2.55 ± 0.05 ^a,b^	2.87 ± 0.17 ^b^	3.67 ± 0.11 ^c^
Cohesiveness	Day 1	0.77 ± 0.02 ^a^	0.74 ± 0.04 ^a^	0.78 ± 0.04 ^a,b^	0.73 ± 0.00 ^a^	0.73 ± 0.03 ^a^
	Day 2	0.62 ± 0.07 ^a^	0.62 ± 0.08 ^a^	0.62 ± 0.01 ^a^	0.59 ± 0.03 ^a^	0.65 ± 0.03 ^a, b^
	Day 3	0.58 ± 0.03 ^a^	0.52 ± 0.15 ^a^	0.58 ± 0.04 ^a^	0.59 ± 0.02 ^a^	0.56 ± 0.02 ^a, b^
Elasticity	Day 1	0.98 ± 0.00 ^a^	0.98 ± 0.00 ^a^	0.98 ± 0.00 ^a^	0.98 ± 0.00 ^a^	0.99 ± 0.02 ^a^
	Day 2	0.98 ± 0.01 ^a^	0.98 ± 0.00 ^a^	0.98 ± 0.00 ^a^	0.99 ± 0.00 ^a, b^	1 ± 0.01 ^b,c^
	Day 3	0.98 ± 0.00 ^a^	0.98 ± 0.00 ^a^	0.98 ± 0.01 ^a^	0.98 ± 0.00 ^a^	0.97 ± 0.02 ^a^
Gumminess (N)	Day 1	0.87 ± 0.03 ^a^	0.94 ± 0.08 ^a^	1.05 ± 0.12 ^a^	1.39 ± 0.13 ^b^	1.55 ± 0.05 ^b^
	Day 2	1.23 ± 0.20 ^a^	1.53 ± 0.19 ^b^	1.52 ± 0.13 ^a,b,c^	1.69 ± 0.19 ^c^	2.12 ± 0.30 ^c^
	Day 3	1.25 ± 0.08 ^a^	0.95 ± 0.20 ^a^	1.45 ± 0.14 ^a,b^	1.68 ± 0.16 ^c^	1.98 ± 0.01 ^b,c^

Note: Data with different letters in the same row are significantly different for *p* < 0.05.

## Data Availability

Data are contained within the article.
